# Rarity in the native range of the Lessepsian migrant *Plocamopherus ocellatus* (Nudibranchia): fact or artifact?

**DOI:** 10.1002/ecy.3481

**Published:** 2021-08-11

**Authors:** Bert W. Hoeksema, Nathalie Yonow

**Affiliations:** ^1^ Naturalis Biodiversity Center P.O. Box 9517 2300 RA Leiden The Netherlands; ^2^ Groningen Institute for Evolutionary Life Sciences University of Groningen P.O. Box 11103 9700 CC Groningen The Netherlands; ^3^ Institute of Biology Leiden Leiden University P.O. Box 9505 2300 RA Leiden The Netherlands; ^4^ Department of Biosciences Swansea University Singleton Park Swansea SA2 8PP United Kingdom

**Keywords:** citizen scientists, Mediterranean Sea, Red Sea, sea slug, Suez Canal

The opening of the Suez Canal in 1869 enabled a large number of Indo‐Pacific marine species to expand their ranges into the Mediterranean Sea (Galil et al. 2017), entering the Gulf of Suez from the Red Sea and migrating northward toward the easternmost basin of the Mediterranean. These species have been called “Lessepsian migrants” (Yonow 2015), “Lessepsian immigrants” (Kleitou et al. 2019), or “Lessepsian invaders” (Ivkić et al. 2019), named after Ferdinand de Lesseps who planned the Suez Canal’s construction, or “Erythraean non‐indigenous species” (Galil et al. 2017), after the Erythraean Sea, an earlier maritime designation including both the Red Sea and the Gulf of Aden.

Among approximately 750 Lessepsian migrants (Galil et al. 2017), there are 30 heterobranchs that constitute ˜6% of the total Mediterranean sea slug fauna (Crocetta et al. 2013). Most are colorful and therefore popular subjects for underwater photographers who have shared numerous observations on internet sites, such as Sea Slug Forum and Mediterranean Slug Site (examples in Appendix S1: Table S1), or directly with sea slug taxonomists (Yonow 2015). This stimulated the use of sea slugs as model organisms in the monitoring of invasive species, in which recreational divers volunteered as citizen scientists (Fernández‐Vilert et al. 2018, Kleitou et al. 2019, Paz‐Sedano et al. 2019).

The nudibranch *Plocamopherus ocellatus* Rüppell & Leuckart, 1828 received increased scientific interest as a Lessepsian migrant when diver observations since the 1980s became the basis for publications (Appendix S1: Table S1). The recent discovery of two small specimens in the Red Sea (Fig. 1) drew attention to the question of whether it was more frequently encountered in the Mediterranean than in its native range (Yonow 2008), and what precisely constitutes this native range.

**Fig. 1:**
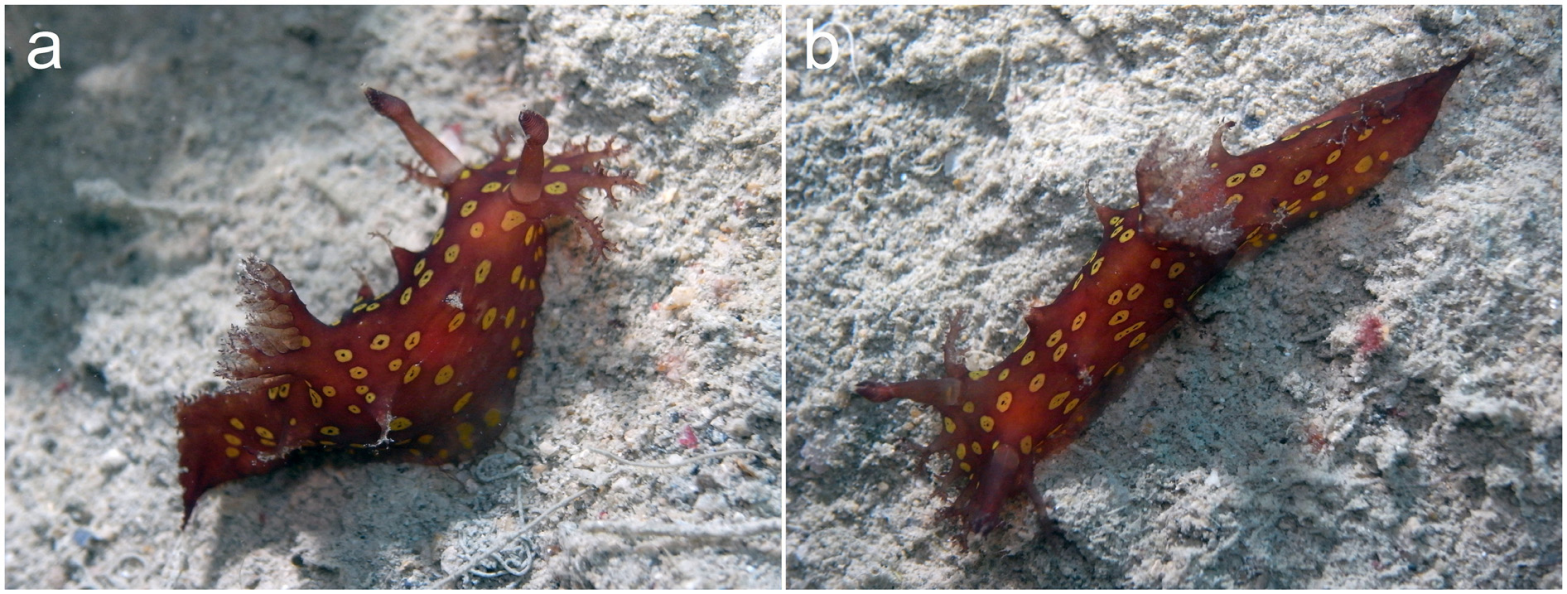
A pair of the “rare” nudibranch *Plocamopherus ocellatus* observed in the Red Sea (Saudi Arabia), Farasan Banks, east side of Safiq Island. (a) One individual (˜20 mm long) is contracted; (b) the other one (˜30 mm long) is extended. Both were crawling together on sand at 27 m depth (5 May 2017) and observed by B. W. Hoeksema during scuba diving.

Indeed, based on records produced by underwater photographers in Israel, Rothman and Galil (2015) concluded that the reputedly rare Erythraean nudibranch *P*. *ocellatus* (see e.g., Nithyanandan 2012) appeared to be not so rare in the eastern Mediterranean. Israel was the first country with a Mediterranean record (1977–2015), followed by observations in Turkey (1998–2019), Lebanon (2000–2015), Cyprus (2015), and Greece (2020) (Fig. 2). The last record is also the westernmost locality: Kastellorizo Island off the Turkish coastline (Ragkousis et al. 2020). Altogether, 23 unique Lessepsian records from 16 localities were found, including two additional museum specimens from the Suez Canal (Appendix S1: Table S1). There are no records from the Mediterranean coast of Egypt or other North African countries.

**Fig. 2:**
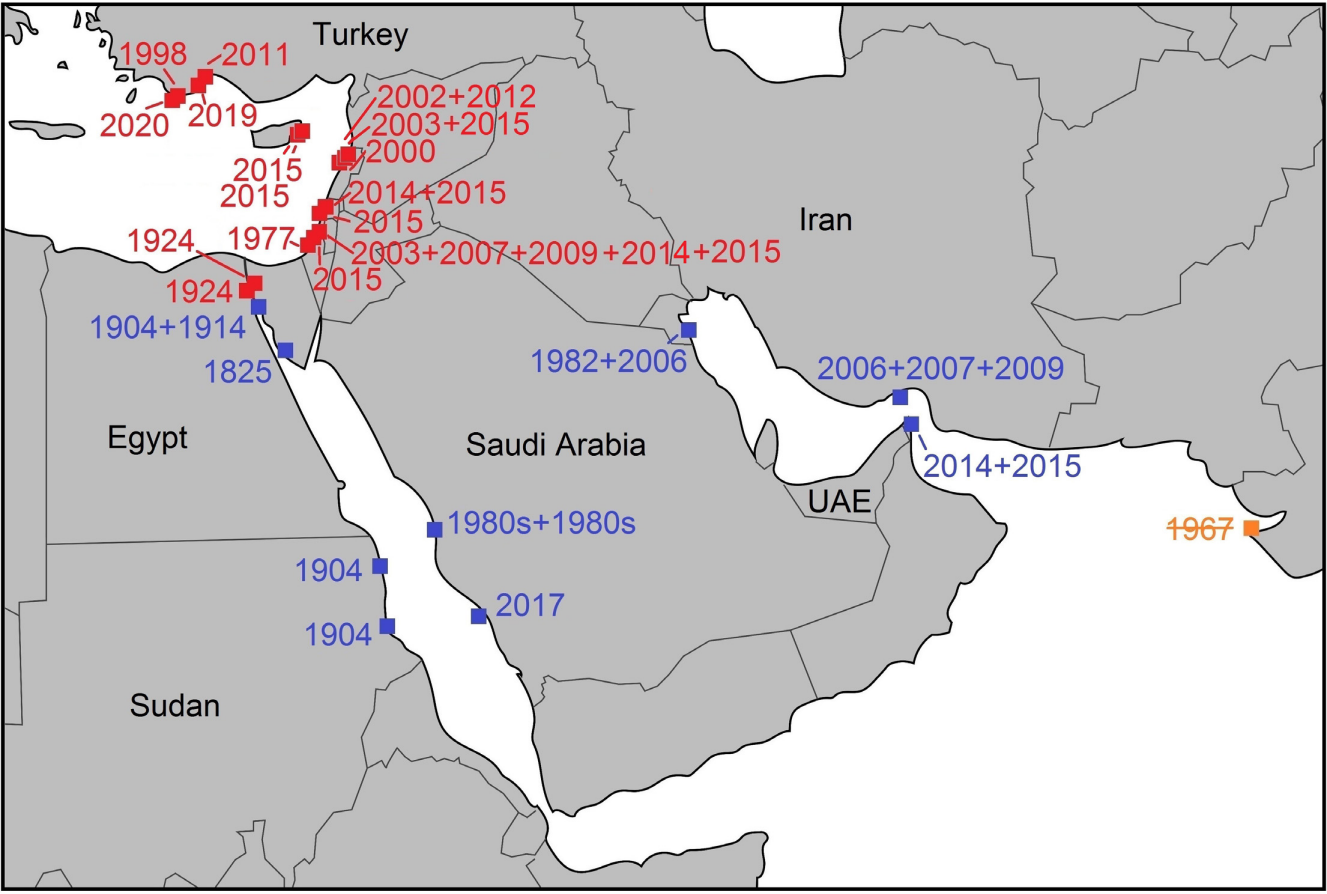
Records (indicated by year) of *Plocamopherus ocellatus* in its native range (blue) and where it was introduced (red), and one erroneous record (orange). Sources: Appendix S1: Table S1.

Only 15 records are known from the native range of *P*. *ocellatus* (Fig. 2). Before the construction of the Suez Canal, the species had only been observed in 1825 at its type locality in the Gulf of Suez. Since then, 14 additional observations at eight localities have been reported in its native range constituting the Gulf of Suez, the Red Sea, the Arabian Gulf, and the Gulf of Oman (Fig. 2). This includes Kuwait in the northern Arabian Gulf, where it appears to be common in March and April (M. Nithyanandan, *personal communication*). No specimens are recorded from the Gulf of Eilat (northeastern Red Sea), despite its well‐developed diving tourism. A specimen record from India (Appendix S1: Table S1) was not included since it is based on a misidentification: its color description does not match with that of *P*. *ocellatus*, which is brownish to dark red or reddish black, with yellow spots that have dark‐colored flecks in the center and a brighter yellow margin followed by a dark ocellation, hence the name (Fig. 1). In poor light conditions, the color is quite cryptic (Rothman and Galil 2015: Fig 1D).

Despite the involvement of citizen scientists, many sea slug species are still rarely encountered. In sea slug ecology, “rarity” is frequently used as a relative and intuitive concept (Schubert and Smith 2020), which has been applied at population or assemblage level (usually expressed as densities) but also in a biogeographical setting (number of locality records) as in the case of *P*. *ocellatus* (e.g., Nithyanandan 2012, Rothman and Galil 2015). Since the opening of the Suez Canal, there are more records (23 from 16 sites) from the Mediterranean than from the much larger native range (14 from eight sites), while underwater photography since the 1980s contributed to 17 records from 14 sites in the non‐native range and 10 records from five sites in the native range (Appendix S1: Table S1). An absence of potential predators in the Mediterranean may play a role, although there is no information on natural predators in the native range. Perhaps attention for invasiveness has encouraged a chase for records in its non‐native range but not in its native range, causing an observer‐expectancy effect. A similar but much more recent case concerns the tiny (<15 mm long) sea slug *Haminoea cyanomarginata* Heller & Thompson, 1983, now synonymized and considered the purple color morph of *Lamprohaminoea ovalis* (Pease, 1868; see Oskars and Malaquias 2020). This minute species has only seven records from its native range (Red Sea, Gulf of Eilat, Gulf of Oman) since its description in 1980 and at least 20 locality records from around the Mediterranean since 2005 (Fernández‐Vilert et al. 2018, Rizgalla et al. 2018). Its maximum length is much smaller than the 60 mm recorded for *P*. *ocellatus* (Ragkousis et al. 2020), which supports the hypothesis that increased awareness can stimulate a quest for new records of introduced species. Nine *P*. *ocellatus* sightings in the Mediterranean were reported from shipwrecks, which are popular among recreational divers and may have contributed to the observer bias mentioned earlier. The diet of *P*. *ocellatus* consists of bryozoans (Yonow 2008, Rothman and Galil 2015), which need a solid substrate and shipwrecks fit in that category, although why some individuals are found on sand is unclear (Fig. 1; Yonow 2008, Rothman and Galil 2015). *Plocamopherus ocellatus* occurs at depths of 1.5–50 m (Appendix S1: Table S1) and produces large egg ribbons attached to solid substrates (Nicolaidou et al. 2012: Fig. 19, Rothman and Galil 2015: Fig. 1C). Although the life history and dispersal mechanisms of *P*. *ocellatus* are not known, spawning and new records indicate its establishment in the Mediterranean. The increasing range and abundance of *P*. *ocellatus* in the Mediterranean could therefore be fact.

On the other hand, among the dense populations of residents and tourists along the Mediterranean shores, there is a large potential of underwater observers. Although the lack of records from the southern Mediterranean and the Gulf of Eilat cannot be explained and needs further investigation, it appears that the rarity in the native range of *P*. *ocellatus* is most likely an artifact.

## Supporting information

Appendix S1
