# Supplementary material for: Rarity in the native range of the Lessepsian migrant Plocamopherus ocellatus (Nudibranchia): fact or artifact?
Source: Ecology. 2021 Aug 11;102(11):e03481. doi: 10.1002/ecy.3481 (PMC9285030; doi:10.1002/ecy.3481)
Supplement: Supplementary file 1 — Appendix S1 [file ECY-102-0-s001.pdf]

Supporting Information. Hoeksema, B.W., and N. Yonow. 2021. Rarity in the native range of the Lessepsian immigrant *Plocamopherus ocellatus* (Nudibranchia): fact or artifact? Ecology.

Appendix S1

Table S1. Records of *Plocamopherus ocellatus* from its native area and where it was introduced, listed in chronological order. Locality data are from the literature, web sites, and original collection (Fig 1). Coordinates are approximate. Years are indicated in blue (native), red (introduced), or orange (misidentified). Depth (m) and substrate are mentioned where known. References per record concern the original observation or collected material, and replicates based on citations or re-use of illustrations. Since the opening of the Suez Canal in 1869, 14 records are native (Gulf of Suez, Red Sea, Arabian Gulf, Gulf of Oman), 22 are introduced (Suez Canal, eastern Mediterranean), and one is misidentified and therefore indicated as an erroneous record (Gulf of Kutch, India).

Locality `	Coordinates (appr.)	Range	Year	Depth	Substrate	Reference(s)
Egypt, Gulf of Suez, Tor	28°13.8'N 33°36.2'E	Native	1825	-	coral	[1] Rüppell and Leuckart (1828), [2] Voigt (1834), [3] Chenu (1859), [4] Issel (1869), [5] Haas (1920), [6] Pruvot-Fol (1933)
Egypt, Gulf of Suez, Suez	29°54.9'N 32°28.0'E	Native	1904	9 m	mud	[7] Eliot (1908)
Sudan, Red Sea, Dongonab	21°01.3'N 37°07.0'E	Native	1904	-	-	[7] Eliot (1908)
Sudan, Red Sea, Suakim	19°05.0'N 37°22.2'E	Native	1904	-	-	[7] Eliot (1908)
Egypt, Gulf of Suez, Suez	29°54.9'N 32°28.0'E	Native	1914	-	-	[8] White (1951), [9] BMNH (2020)
Egypt, Suez Canal, Toussoum	30°29.7'N 32°20.5'E	Introduced	1924	-	-	[10] O’Donoghue (1929), [11] BMNH (2020)
Egypt, Suez Canal, Le Deservoir	30°35.8'N 32°18.7'E	Introduced	1924	-	-	[10] O’Donoghue (1929), [12] BMNH (2020)

India, Gulf of Kutch, Okha	22°28.6'N 69°03.8'E	Misidentified	1967	-	-	[13] Narayanan (1968), [14] Rao and Sastry (2005), [15] Venkataraman et al. (2015), [16] Raghunathan et al. (2016), [17] Vadher et al. (2020)
Israel, Medit., Nizanim	31°44.2'N 34°35.2'E	Introduced	1977	50 m	-	[18–20] Barash and Danin (1982, 1986, 1992)
Saudi Arabia, Red Sea, Jeddah	21°44'N 39°00'E	Native	1980s	-	-	[21] J. Kuchinke, photographic record in Lieske and Myers (2004), [22] idem in Yonow (2008)
Saudi Arabia, Red Sea, Jeddah	21°44'N 39°00'E	Native	1980s	-	-	[23] W. Pridgen, photographic record (unpublished, N. Yonow, pers. comm.)
Kuwait, Persian Gulf, Al-Khiran	28°40.6'N 48°21.5'E	Native	1982	-	rock pool	[24] Jones (1986), [25] Al-Kandari et al. (2020)
Turkey, Medit., Kas	36°11.9'N 29°38.3'E	Introduced	1998	8–10 m	rock	[26] Byukbaykal (2002), [27] Koehler (2002), [28–29] Rudman (2002a, 2002b), [30] Yokeş and Rudman (2004), [31] Debelius and Kuiter (2007), [32] Gosliner et al. (2008), [33] Bielecki (2011), [34] Tzomos et al. (2012), [35] Yokeş et al. (2012), [36] Öztürk et al. (2014), [37] Gosliner et al. (2015, 2018), [38] Çevik and Gündoğdu (2016), [39] Öztürk et al. (2017)
Lebanon, Medit., Chak El Hatab	34°17.7'N 35°40.2'E	Introduced	2000	5 m	-	[40] Valdes and Templado (2002), [41] Zibrowius and Bitar (2003), [42] Crocetta et al. (2013), [43] Bitar (2014), [44] Crocetta et al.(2020)
Lebanon, Medit., Raoucheh	33°53.4'N 35°28.2'E	Introduced	2002	7 m	limestone rock	[40–44]
Lebanon, Medit., Beirut	33°53.1'N 35°28.5'E	Introduced	2003	15 m	breakwater	[42–44]
Israel, Medit., Jaffa (Tel Aviv)	32°03.8'N 34°43.9'E	Introduced	2003	27 m	shipwreck	[45] Levin (2003), [46] Rothman and Galil (2015)
Kuwait, Persian Gulf, Al-Khiran	28°40.6'N 48°21.5'E	Native	2006	1.5 m	marina wall	[47] Nithyanandan (2012), [25] Al-Kandari et al. (2020)
Iran, Gulf of Oman, Qeshm Isl.	26°59.8'N 56°12.1'E	Native	2006	-	-	[48] M.S. Ranjbar, photo in Trainito and Doneddu (2014)

Iran, Gulf of Oman, Qeshm Isl.	26°59.8'N 56°12.1'E	Native	2007	2–5 m	reef rock	[49] Moradi (2010a), Rudman (2010a), [50] Rezai et al. (2016)
Israel, Medit., Jaffa (Tel Aviv)	32°03.8'N 34°43.9'E	Introduced	2007	25 m	shipwreck	[46] Rothman and Galil (2015)
Iran, Gulf of Oman, Qeshm Isl.	26°59.8'N 56°12.1'E	Native	2009	2.5 m	reef rock	[51] Moradi (2010b), Rudman (2010b) [50] Rezai et al. (2016)
Israel, Medit., Jaffa (Tel Aviv)	32°03.8'N 34°43.9'E	Introduced	2009	25 m	shipwreck	[46] Rothman and Galil (2015)
Turkey, Medit., Iskenderun Bay	36°49.8'N 35°53.0'E	Introduced	2011	4 m	rock	[52] Nicolaidou et al. (2012)
Lebanon, Medit., Raoucheh	33°53.4'N 35°28.2'E	Introduced	2012	-	limestone rock	[53] Ramos-Esplá et al. (2015)
UAE, Gulf of Oman, Fujairah	25°06.7'N 56°22.1'E	Native	2014	-	rock	[54] Petkovic (2014)
Israel, Medit., Achziv	33°02.4'N 35°04.4'E	Introduced	2014	26 m	shipwreck	[46] Rothman and Galil (2015)
Israel, Medit., Jaffa (Tel Aviv)	32°03.8'N 34°43.9'E	Introduced	2014	32 m	shipwreck	[55] Amar (2014)
Cyprus, Medit., Proteras	35°00.2'N 34°04.2'E	Introduced	2015	25 m	shipwreck	[56] Crocetta et al. (2015)
Cyprus, Medit., Proteras	34°58.5'N 33°58.4'E	Introduced	2015	19 m	shipwreck	[56] Crocetta et al. (2015)
Israel, Medit., Achziv	33°02.4'N 35°04.4'E	Introduced	2015	26 m	shipwreck	[46] Rothman and Galil (2015)
Israel, Medit., Jaffa (Tel Aviv)	32°03.8'N 34°43.9'E	Introduced	2015	25 m	shipwreck	[46] Rothman and Galil (2015)
Israel, Medit., Nahariya	33°01.5'N 35°05.5'E	Introduced	2015	30 m	shipwreck	[46] Rothman and Galil (2015)
Israel, Medit., Ashdod Port	31°49.0'N 34°38.4'E	Introduced	2015	15 m	breakwater	[46] Rothman and Galil (2015)
Lebanon, Medit., Beirut	33°53.1'N 35°28.5'E	Introduced	2015	-	-	[57] Ballasteros et al. (2014)
UAE, Gulf of Oman, Fujairah	25°06.7'N 56°22.1'E	Native	2016	-	fish cage	[58,59] Clarke (2016, 2020)
Saudi Arabia, Red Sea, Farasan Banks	19°32.4'N 40°65.0'E	Native	2017	27 m	mud / sand	[60] Present study (Fig. 1)
Turkey, Medit., Phaselis	36°32.4'N 30°33.8'E	Introduced	2019	7 m	rock	[61] Gökoğlu et al. (2019)
Greece, Medit., Kastellorizo Is.	36°07.6'N 29°34.7'E	Introduced	2020	12 m	rock, cave	[62] Ragkousis et al. (2020)

References

1. Rüppell, E., and F.S. Leuckart. 1828. Mollusca. Atlas zu des Reise im Nordlichen Afrika von Eduard Rüppell. 1. Abth. Zoologie. 5. Neue wirbellose Thiere des Rothen Meers. H.L. Brönner, Frankfurt am Main, Germany, 22 pp., 12 pls.
2. Voigt, F.S. 1834. Die Mollusken. Das Thierreich geordnet nach seiner Organisation. Als Grundlage der Naturgeschichte der Thiere und Einleitung in die vergleichende Anatomie vom Baron du Cuvier. Dritter Band. F.A. Brockhaus, Leipzig, 621 pp.
3. Chenu, J.C. 1859. Manuel de conchyliologie et de paléontologie conchyliologique. Tome 1. Masson, Paris, France, vii + 508 pp.
4. Issel, A. 1869. Malacologia del mar rosso, ricerche zoologiche e paleontologiche di Arturo Issel: Memoria letta al congresso dei Naturalisti Italiani in Vicenza nel 1868. Pisa, Editori della Biblioteca Malacologica: Pisa, Italy, xi + 388 pp., 5 pls. https://ia800208.us.archive.org/19/items/malacologiadelma00isse/malacologiadelma00isse.pdf
5. Haas, F. 1920. Opistobranchier aus verschiedenen warmen Meeren. Archiv für Molluskenkunde 52:138–142.
6. Pruvot-Fol, A. 1933. Mission Robert Ph. Dollfus en Égypte. Opisthobranchiata. Mémoires de l'Institut d' Égypte 21:89–159, pls. 1–4
7. Eliot, C. 1908. Reports on the marine biology of the Sudanese Red Sea. XI: Notes on a collection of nudibranchs from the Red Sea. Journal of the Linnean Society London, Zoology 31:86–122. https://doi.org/10.1111/j.1096-3642.1908.tb00457.x
8. White, K.M. 1951. On a collection of molluscs, mainly nudibranchs, from the Red Sea. Proceedings of the Malacological Society, London 28:241–253. https://doi.org/10.1093/oxfordjournals.mollus.a064590
9. Locality record. BMNH 1914.1.8.210-211. https://data.nhm.ac.uk/object/8e1a028d-dc2c-460b-9e09-861a60289fa9. Accessed: 29/11/2020.
10. O'Donoghue, C.H. 1929. Report on the Opisthobranchia. In: Zoological results of the Cambridge Expedition to the Suez Canal, 1924. Transactions of the Zoological Society of London 22:713–841. https://doi.org/10.1111/j.1096-3642.1929.tb00212.x
11. Locality record. BMNH 1933.6.30.47. https://data.nhm.ac.uk/object/bbbfc219-4bb0-46bf-8d36-8fc042d4328d. Accessed: 29/11/2020.
12. Locality record. BMNH 1933.6.30.45–46. https://data.nhm.ac.uk/object/08ea2bb9-c078-4983-9bbb-35d25ac07da5. Accessed: 29/11/2020.
13. Narayanan, K.R. 1968. On the Opisthobranchiate fauna of the Gulf of Kutch. Proceedings of the Symposium on Mollusca held at Cochin from January 12 to 16, 1968, Symposium Series 3, 1:189–213. Marine Biological Association of India, Mandapam.
14. Rao, N.V.S., and D.R.K. Sastry. 2005. Fauna of Marine National Park, Gulf of Kachchh (Gujarat). An overview. Conservation Area Series 23, 79 pp., 8 pls. Zoological Survey of India, Kolkata, India. http://faunaofindia.nic.in/PDFVolumes/cas/023/index.pdf
15. Venkataraman, K., C. Raghunathan, R. Raghuraman, and S. Dixit. 2015. Fascinating Seaslugs & Flatworms of Indian Seas, Zoological Survey of India, Kolkata, India, 149 pp. https://www.researchgate.net/publication/303971346_Fascinating_Seaslugs_and_flatworms_of_Indian_Seas
16. Raghunathan, C., S. Dixit, and K. Chandra. 2016. Opisthobranchs in coastal waters of India, pp. 229–246. *In* K. Chandra, C. Raghunathan, T. Mondal, and S. Dash, eds. Current Status of Marine Faunal Diversity in India. Zoological Survey of India, Kolkata, India. https://www.researchgate.net/publication/317646000_Current_Status_of_Marine_Faunal_Diversity_in_India

17. Vadher, P., H. Kardani, and I. Beleem. 2020. An annotated checklist of sea slug fauna of Gujarat coast, India. Journal of Threatened Taxa 12:15835–15851. https://doi.org/10.11609/jott.5278.12.8.15835-15851
18. Barash, A., and Z. Danin. 1982. Mediterranean Mollusca of Israel and Sinai; composition and distribution. Israel Journal of Zoology 31:86–118. https://doi.org/10.1080/00212210.1982.10688524
19. Barash, A., and Z. Danin. 1986. Further additions to the knowledge of Indo-Pacific Mollusca in the Mediterranean Sea. Spixiana 9:117–141. https://www.biodiversitylibrary.org/page/28256846#page/127/mode/1up
20. Barash, A., and Z. Danin. 1992. Fauna Palaestina: Mollusca I. Annotated list of Mediterranean molluscs of Israel and Sinai. The Israel Academy of Sciences and Humanities, Jerusalem, 405 pp.
21. Kuchinke, J. 1980s. Photographic record as *Plocamopherus* sp. in Lieske and Myers (2004, p. 300). Lieske, E., and R.F. Myers. 2004. Coral reef guide Red Sea to Gulf of Aden, south Oman. Harper Collins Publishers, London, 384 pp.
22. Kuchinke, J. 1980s. Photographic record in Yonow, N. 2008. Sea slugs of the Red Sea. Pensoft Publishers, Sophia and Moscow.
23. Pridgen, W. 1980s. Photographic record (unpublished, Yonow personal communication)
24. Jones, D.A. 1986. A field guide to the sea shores of Kuwait and the Arabian Gulf, University of Kuwait. Blandford Press, Dorset, U.K. Collection data (personal communication D.A. Jones, 22/12/2020).
25. Al-Kandari, M., P.G. Oliver, W. Chen, V. Skryabin, M. Raghu, A. Yousif, S. Al-Jazzaf, A. Taqi, and A. AlHamad. 2020. Diversity and distribution of the intertidal Mollusca of the State of Kuwait, Arabian Gulf. Regional Studies in Marine Science 33:100905. https://doi.org/10.1016/j.rsma.2019.100905
26. Byukbaykal, F. 2002. *Plocamopherus ocellatus* Rüppell & Leuckart, 1828. Mediterranean Slug Site. http://www.medslugs.de/E/Med/Plocamopherus_ocellatus/Plocamopherus_ocellatus_01.htm. Accessed: 23/12/2020.
27. Koehler, E. 2002 (Nov 27) *Plocamopherus ocellatus* from Turkey. [Message in] Sea SlugForum. Australian Museum, Sydney. Available from http://www.seaslugforum.net/find/8516. Accessed: 29/11/2020.
28. Rudman, W.B. 2002 (Nov 26) *Plocamopherus ocellatus* Rueppell & Leuckart, 1828. [In] Sea Slug Forum.Australian Museum, Sydney. Available from http://www.seaslugforum.net/find/plococel. Accessed: 29/11/2020.
29. Rudman, W.B. 2002 (Nov 27). Comment on *Plocamopherus ocellatus* from Turkey by Erwin Koehler. [Message in] Sea Slug Forum. Australian Museum, Sydney.Available from http://www.seaslugforum.net/find/8516. Accessed: 29/11/2020.
30. Yokeş, M.B., and W.B. Rudman. 2004. Lessepsian opisthobranchs from southwestern coast of Turkey; five new records for Mediterranean. Rapport de la Commission Internationale pour l'Exploration Scientifique de la Mer Méditerranée 37:557.
31. Debelius, H., and R.H. Kuiter. 2007. Nudibranchs of the World. IKAN-Unterwasserarhiv, Frankfurt, Germany, 362 pp.
32. Gosliner, T.M., D.W. Behrens, and A. Valdés. 2008. Indo-Pacific nudibranchs and sea slugs. A field guide to the Worlds'most diverse fauna. Sea Challengers Natural History Books, Gig Harbor, and California Academy of Sciences, San Francisco, U.S.A. 426 pp.
33. Bielecki, S., G. Cavignaux, J.M. Crouzet, and S. Grall. 2011. Des limaces de rêve - Opistobranches de Méditerranée. Sandrine Bielecki, Peymeinade, France, 249 pp.

34. Tzomos, T., M.-S. Kitsos, D. Koutsoubas, and A. Koukouras. 2012. Evolution of the entrance rate and of the spatiotemporal distribution of Lessepsian Mollusca in the Mediterranean Sea. Journal of Biological Research Thessaloniki 17:81–96. https://media.proquest.com/media/pq/classic/doc/2673518121/fmt/pi/rep/NONE?_s=odVKmVA2a8svBw9hWhzQM0NcxCk%3D
35. Yokeş, M.B., C. Dalyan, S.Ü. Karhan, V. Demir, U. Tural, and E. Kalkan. 2012. Alien opisthobranchs from Turkish coasts: first record of *Plocamopherus tilesii* Bergh, 1877 from the Mediterranean. Triton 5:1–9. https://www.researchgate.net/publication/253644931_Alien_opisthobranchs_from_Turkish_coasts_first_record_of_Plocamopherus_tilesii_Bergh_1877_from_the_Mediterranean
36. Öztürk, B., A. Doğan, B.B. Bakır, and A. Salman. 2014. Marine molluscs of the Turkish coasts: an updated checklist. Turkish Journal of Zoology 38:832–879. https://journals.tubitak.gov.tr/zoology/issues/zoo-14-38-6/zoo-38-6-7-1405-78.pdf
37. Gosliner, T.M., A. Valdés, and D.W. Behrens. 2015 [2018]. Nudibranch & sea slug identification Indo-Pacific [2nd edition]. New World Publications, Jacksonville, Fl., USA, 451 pp.
38. Çevik, C., and S. Gündoğdu. 2016. Marine molluscs of Mediterranean Coast of Turkey. *In* C. Turan, B. Salihoğlu, E. Özgür Özbek, B. Öztürk (eds.) The Turkish part of the Mediterranean Sea. Marine biodiversity, fisheries, conservation and governance. Turkish Marine Research Foundation (TUDAV), Publication No: 43, Istanbul, Turkey, pp. 184–197. http://tudav.org/wp-content/uploads/2018/04/MEDITERRANEAN_SEA_2016.pdf
39. Öztürk, B., B. Bitlis, A. Doğan, and N. Türkçü. 2017. Alien marine molluscs along the Turkish coast, with a new record of *Varicopeza pauxilla* (A. Adams, 1855) (Mollusca: Gastropoda) from the Mediterranean Sea. Acta Zoologica Bulgarica Suppl. 9:83–92.
40. Valdés, A., and J. Templado. 2002. Indo-Pacific dorid nudibranchs collected in Lebanon (eastern Mediterranean). Iberus 20:23–30. https://www.researchgate.net/publication/292116392_Indo-Pacific_dorid_nudibranchs_collected_in_Lebanon_eastern_Mediterranean
41. Zibrowius, H., and G. Bitar. 2003.Invertébrés marins exotiques sur la côte du Liban. Lebanese Science Journal 4:67–74. http://lsj.cnrs.edu.lb/wp-content/uploads/2016/01/zibrowius.pdf
42. Crocetta, F., H. Zibrowius, G. Bitar, J. Templado, and M. Oliverio. 2013. Biogeographical homogeneity in the eastern Mediterranean Sea - I: the opisthobranchs (Mollusca: Gastropoda) from Lebanon. Mediterranean Marine Science 14:403–408. (Supplementary data) http://dx.doi.org/10.12681/mms.404
43. Bitar, G. 2014. Les mollusques exotiques de la côte Libanaise. Bulletin de la Société zoologique de France 139:37–45. https://societe-zoologique.fr/sites/default/files/revue/2017-10/Z139Bitar.pdf
44. Crocetta, F., G. Bitar, H. Zibrowius, and M. Oliverio. 2020. Increase in knowledge of the marine gastropod fauna of Lebanon since the 19th century. Bulletin of Marine Science 96:1–22. https://doi.org/10.5343/bms.2019.0012
45. Levin, M. 2003. *Plocamopherus ocellatus* from Israel. [Message in] Sea Slug Forum. Sydney: Australian Museum. Available at: http://www.seaslugforum.net/showall/plococel. Accessed: 29/11/2020.
46. Rothman, S.B.-S., and B.S. Galil. 2015. Not so rare: *Plocamopherus ocellatus* (Nudibranchia, Polyceridae) in the Eastern Mediterranean. Marine Biodiversity Records 8:e144. https://doi.org/10.1017/S1755267215001207

47. Nithyanandan, M. 2012. New and rare nudibranch records from Kuwait, Arabian Gulf (Mollusca: Opisthobranchia). Marine Biodiversity Records 5:e115. https://doi.org/10.1017/S1755267212000954
48. M.S. Ranjbar, photographic record in Trainito, E., and M. Doneddu. 2014. Nudibranchi del Mediterraneo, 2nd Edition. Il Castello, Milano, Italy,191 pp. (date of photograph 23/04/2006: personal communication E. Trainito, 01/01/2021)
49. Moradi, M. 2010a. *Plocamopherus ocellatus* from Iran [2]. [Message in] Sea Slug Forum. Australian Museum, Sydney. Available from http://www.seaslugforum.net/find/23602. Accessed: 29/11/2020; Rudman, W.B. 2010a (May 4). Comment on *Plocamopherus ocellatus* from Iran [2] by Mahdi Moradi Och Tapeh. [Message in] Sea Slug Forum. Australian Museum, Sydney. Available from http://www.seaslugforum.net/find/23602. Accessed: 29/11/2020.
50. Moradi, M. 2010b. *Plocamopherus ocellatus* from Iran [1]. [Message in] Sea Slug Forum. Australian Museum, Sydney. Available from http://www.seaslugforum.net/find/23601. Accessed: 29/11/2020; Rudman, W.B. 2010b (May 4). Comment on *Plocamopherus ocellatus* from Iran [1] by Mahdi Moradi Och Tapeh. [Message in] Sea Slug Forum. Australian Museum, Sydney. Available from http://www.seaslugforum.net/find/23601. Accessed: 29/11/2020.
51. Rezei, H., S.A. Mohtarami, H. Dehghani, P. Tavakoli-Kolour, H.R. Bargahi, and K. Kabiri. 2016. Nudibranchs from the northern Persian Gulf. Journal of the Persian Gulf 7:71–78. http://aquaticcommons.org/26105/1/JPG-v7n0p71-en.pdf
52. Nicolaidou, A., G. Alongi, O. Aydogan, M. Catra, et al. 2012. New Mediterranean Biodiversity Records (June 2012). Mediterranean Marine Science 13:162–174. https://doi.org/10.12681/mms.33
53. Ramos-Esplá, A.A., G. Bitar, G. Khalaf, H. El Shaer, A. Forcada, A. Limam, O. Ocaña, Y.R. Sghaier, and C Valle. 2015. Ecological characterization of sites of interest for conservation in Lebanon: Enfeh Peninsula, Ras Chekaa cliffs, Raoucheh, Saida, Tyre and Nakoura. Ed. RAC/SPA - MedMPAnet Project, Tunis. 216 pp.
54. Petkovic, D. 2014. *Plocamopherus ocellatus* Rüppell & Leuckart, 1828. Mediterranean Slug Site. http://www.medslugs.de/E/Ind-NW/Plocamopherus_ocellatus/Plocamopherus_ocellatus_02.htm. Accessed: 23/12/2020.
55. Amar, F. 2014. *Plocamopherus ocellatus*. Mediterranean Sea, Tel Aviv, Israel 2014. https://www.rafiamar.com/nudibranch1. Accessed: 03/03/2021.
56. Crocetta, F., D. Agius, P. Balistreri, M. Bariche, et al. 2015. New Mediterranean Biodiversity Records (October 2015). Mediterranean Marine Science 16:682–702. https://doi.org/10.12681/mms.1477
57. Ballesteros, M., E. Madrenas, and M. Pontes. 2014. *Plocamopherus ocellatus* in OPK-Opistobranquis. Published: 19/09/2019. https://opistobranquis.info/en/guia/nudibranchia/doridina/doridoidei/polyceroidea/plocamopherus-ocellatus. Accessed: 29/11/2020.
58. Clarke, S. 2016. *Plocamopherus ocellatus* Rüppell & Leuckart, 1828. Mediterranean Slug Site. http://www.medslugs.de/E/Ind-NW/Plocamopherus_ocellatus/Plocamopherus_ocellatus_03.htm. Accessed: 23/12/2020.
59. Clarke, S. 2020. *Plocamopherus ocellatus*. iNaturalist. https://www.inaturalist.org/observations/41665405. Accessed: 17/12/2020.
60. Hoeksema, B.W. pers. obs. (Fig. 1) two specimens of *Plocamopherus ocellatus* observed while scuba diving in the southern Red Sea (Saudi Arabia), east side of Safiq Isl., on sand, 27 m depth, 5 May 2017. KAUST Farasan Banks Cruise, 1–7 May 2017, King Abdullah University

of Science and Technology, Thuwal, Saudi Arabia. The specimens have been deposited in the mollusc collection of Naturalis Biodiversity Center, Leiden, with catalogue numbers RMNH.MOL.342679–342680.

61. Gökoğlu, M., S. Teker, J. Korun, and K. Gökoğlu. 2019. A Lesepsiyan opisthobranch (*Plocamopherus ocellatus* Rüppell & Leuckart, 1828) in the shore of Phaselis ancient city. Phaselis 5:125–128. http://dx.doi.org/10.18367/Pha.19006
62. Ragkousis, M, N. Abdelali, E. Azzurro, A. Badreddine, et al. 2020. New alien Mediterranean biodiversity records (October 2020). Mediterranean Marine Science 21:631–652. https://doi.org/10.12681/mms.23673
